# Foreign bodies ingestion

**DOI:** 10.11604/pamj.2020.35.96.21356

**Published:** 2020-04-01

**Authors:** Danilo Coco, Silvana Leanza

**Affiliations:** 1 Ospedali Riuniti Marche Nord, Pesaro, Italy; 2 Carlo Urbani Hospital, Jesi, Ancona, Italy

**Keywords:** Foreign bodies ingestion, key, Italy

## Image in medicine

Foreign body ingestion (FB) obstruction or impaction depend on the physical properties of the object, including size, shape and composition. Foreign body ingestion and food bolus impaction occur commonly. The majority of ingested foreign bodies will pass spontaneously. Pre-endoscopic series have shown that 80% or more of foreign objects will likely pass without the need for intervention. A 40-year-old Caucasian man was admitted to Emergency Room of our institution for acute abdominal pain. His past medical history was negative for previous gastrointestinal disease or surgery. He was on medical therapy for schizophrenia. He not referred any acute abdominal pain saying he has eaten a number of foreign object 6 hours before to admission. His abdominal physical examination was unremarkable. White blood tests were in normal range. Body temperature was 36.2^o^C. Chest X-ray was normal. Abdominal X-ray not showed free subdiaphragmatic air but showed five thin foreign bodies like a pins and a key in right iliac fossa. The patient was referred to gastrointestinal unit only to be observed. At the 6-day follow-up, the patient was in good clinical condition, blood tests were normal, and bowel function was recovered.

**Figure 1:**
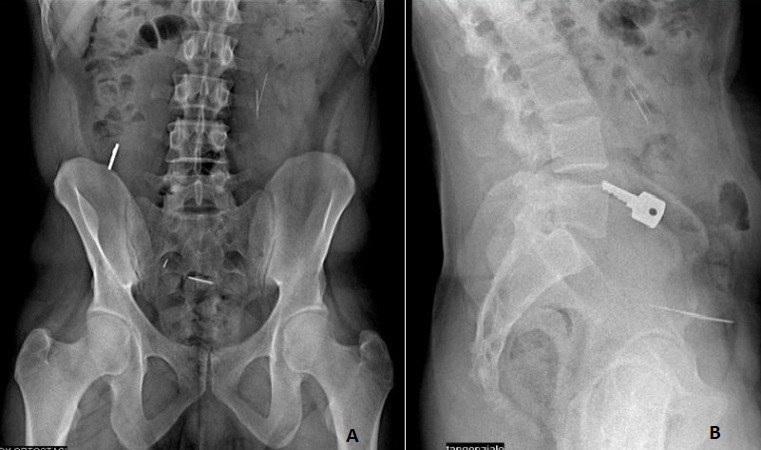
(A) foreign bodies: brooches; (B) foreign bodies: key

